# The aMAP Score is an Independent Risk Factor for Intermediate-stage Hepatocellular Carcinoma: A Large Retrospective Cohort Study

**DOI:** 10.7150/jca.79377

**Published:** 2023-05-08

**Authors:** Yaying Chen, Yanhong Shi, Linbin Lu, Xuewen Wang, Qin Lin, Yihong Lin, Ruiqi Wang, Hongwu Zhu, Peichan Zheng, Xiong Chen

**Affiliations:** 1 Department of Oncology, Mengchao Hepatobiliary Hospital of Fujian Medical University, Fuzhou, Fujian, China.; 2 Department of Oncology, The 900th Hospital of the People's Liberation Army Joint Service Support Force, Fuzong Clinical Medical College of Fujian Medical University, Fuzhou, Fujian, China.; 3 Department of Gastroenterology, Xiamen Humanity Hospital, Xiamen, China.; 4 Department of Gastroenterology, General Hospital of Southern Theater Command, People's Liberation Army of China, Guangzhou, Fujian, China.; 5 Fujian Key Laboratory of Drug Target Discovery and Structural and Functional Research, Fuzhou, China.; 6 Fujian Center for Safety Evaluation of New Drug, Fujian Medical University, Fuzhou, China.

**Keywords:** hepatocellular carcinoma, intermediate-stage HCC, BCLC stage B, overall survival, aMAP score, predicted model, nomogram, TACE

## Abstract

**Background**: A less effective nomogram for patients with intermediate-stage hepatocellular carcinoma (HCC) to predict overall survival (OS) is available. This study aimed to investigate the role of age-male-albumin-bilirubin-platelet (aMAP) scores in the prognosis of patients with intermediate-stage HCC and develop an aMAP score-based nomogram to predict OS.

**Methods**: Data on newly diagnosed intermediate-stage patients with HCC at Sun Yat-sen University Cancer Center between January 2007 and May 2012 were retrospectively collected. Independent risk factors affecting prognosis were selected by multivariate analyses. The optimal cut-off value for the aMAP score was determined using X-tile. The survival prognostic models were presented by the nomogram.

**Results**: For the 875 patients with intermediate-stage HCC included, the median OS was 22.2 months (95% CI 19.6-25.1). Patients were classified into three groups by X-tile plots (aMAP score < 49.42; 49.42 ≤ aMAP score < 56; aMAP score ≥ 56). Alpha-fetoprotein, lactate dehydrogenase, aMAP score, diameter of main tumor, number of intrahepatic lesions, and treatment regimen were independent risk factors for prognosis. A predicted model was constructed with a C-index of 0.70 (95% CI: 0.68-0.72) in the training goup, and its 1-, 3-, and 5-year area under the receiver operating curve were: 0.75, 0.73, and 0.72. The validation group of the C-index is 0.82. Calibration graphs showed good consistency between the actual and predicted survival rates. The decision curve analysis suggested the clinical utility of the model, which may help clinicians guide clinical decision-making.

**Conclusion**: The aMAP score was an independent risk factor for intermediate-stage HCC. The aMAP score-based nomogram has good discrimination, calibration, and clinical utility.

## Introduction

Liver cancer is the sixth most common tumor worldwide. Hepatocellular carcinoma (HCC) accounts for 75%-85% of liver cancer diagnoses 1. The prognosis of HCC is not only affected by the size of the tumor burden but also by the residual liver function. In China, most patients have associated hepatitis B-related disease or concomitant *Aspergillus flavus* infection 2.

Intermediate-stage HCC, also known as Barcelona Clinic Liver Cancer (BCLC) stage B, is the most extensive stage of BCLC 3. This group of patients may vary in terms of liver function and tumor burden, with a high Child-Pugh score of 5-9 points, significant differences in tumor size, varying tumor number, and distribution in one or two lobes, resulting in significant differences in the survival prognosis of patients 4. The clinical benefit assessed by traditional means often differs somewhat from the actual benefit. Patients with better liver function and smaller tumor burden have a more optimistic prognosis. Studies have shown that for patients with fair liver function and small tumor burden, the 2-year overall survival rate may reach up to 63% 5.

Risk prediction models have been widely used in lung cancer, gastrointestinal tumors, and prostate cancer, among others. 6, 7. A variety of models that predict the survival or recurrence rate of liver cancer have been established 8, 9. Factors such as albumin, bilirubin, platelets, alpha-fetoprotein (AFP), tumor location, tumor number, and microvascular invasion can affect the prognosis of HCC 10, 11. Thus, subsequent studies have adjusted for these risk factors to construct different models. The most widely known staging systems include the Okuda staging system, Child-Pugh liver function grading criteria, TNM staging system, Italian Liver Cancer Program (CLIP), and BCLC staging systems. However, the performance of these staging systems is unsatisfactory because of their low applicability in distinguishing heterogeneous cohorts. Therefore, many newly developed scoring systems, such as albumin-bilirubin (ALBI), platelet-albumin-bilirubin (PALBI), ALBI-T, and modified ALBI-T, have been developed 12, 13. These scoring systems mainly use laboratory indicators such as albumin, bilirubin, platelets, and AFP, show superior predictive ability to more specifically predict the prognosis of individual patients, and affirm the irreplaceable role of clinical laboratory indicators in prognosis prediction. However, none of these models are suitable for patients with intermediate-stage HCC. There is still a lack of survival prognostic models for patients with intermediate-stage HCC.

Recently, Hou 14 developed an age-male-ALBI-platelet (aMAP) scoring system - the world's first diagnostic prediction model for predicting the 5-year risk of HCC across diseases and ethnic groups. They verified that five parameters, including age, sex, platelets, albumin, and bilirubin, were independent risk factors for the development of HCC. The patients were divided into three groups by aMAP scores: high risk, middle risk, and low risk groups (high risk group: aMAP score > 60; middle risk group: 50 ≤ aMAP score ≤ 60; low risk group: aMAP score < 50).

There are several prediction models based on aMAP scores, which are mostly used for predicting the likelihood of liver disease patients with different baseline characteristics developing HCC 15-17. In addition, aMAP score as the primary variable was constructed to predict recurrence of HCC after radiofrequency ablation in a Chinese population prediction model by two other studies 18, 19. Studies have shown that there is a certain relationship between the chronic development of liver cancer and the survival prognosis of patients 20.

The importance of the aMAP score in assessing the OS of intermediate-stage HCC has not been determined. Therefore, we retrospectively collected data from HCC patients to investigate the risk factors affecting OS and constructed a new model based on the aMAP score specialized for intermediate-stage HCC patients.

## Materials and Methods

### Data sources and patient selection

The clinical data of this study were mainly derived from a large retrospective multicenter study with a detailed introduction 21-24. In this study, we included the baseline clinical data of intermediate-stage HCC patients in the derivation cohort at Sun Yat-sen University Cancer Center from January 2007 to May 2012 who received TACE or hepatectomy as first-line treatment.Validation cohort was established by IM-HCC patients from multiple centre between December 2018 to May 2022.Patients were followed up monthly during the period of initial treatment for the first 2 years while decreasing to every 3-6 months after 2 years of remission.

Patients who satisfied the inclusion criteria were enrolled and excluded if they met any of the exclusion criteria. The inclusion criteria were as follows: clinical diagnosis of stage B HCC; complete data any of the following at initial diagnosis (computerized tomography (CT) or magnetic resonance imaging of the abdominal region, radiography or CT of the chest, routine bloodwork test, biochemical routine test, serum AFP level, and coagulation indices); chronic hepatitis B virus-associated hepatocellular carcinoma; no history of other malignancies. The exclusion criteria were as follows: absence of laboratory parameters such as albumin (ALB), platelet count, and TBIL at initial diagnosis.

The Clinical Research Department of the Sun Yat-sen University Cancer Center approved the study protocol (2017-FXY-129). Since this study was a secondary analysis study and the patient data were anonymized, the need for informed consent was waived. Patients or the public were not involved in our study's design, conduct, reporting, or dissemination plan.

### Definitions of variables and outcomes

Only baseline data of serum tumor markers, medical imaging, and biochemical and hematological parameters were included in the analysis. The primary site of the tumor in both the left and right lobes was defined as both lesions. aMAP score was calculated using sex, age, total bilirubin (μmol/L), albumin (g/L), and platelets (103/mm^3^). The formula 14 is as follows:



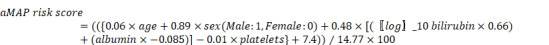



The primary study endpoint of this study was overall survival (OS).

### Model construction, performance evaluation, and statistical analysis

The optimal cut-off value of the aMAP score and the model risk score were determined using X-tile software 25. Categorical variables are presented as numbers and proportions. In this study, the Kaplan-Meier method was used to calculate the OS, and the log-rank test was used to compare the differences. Factors affecting OS were identified using the Cox proportional hazards model. Variables with p < 0.05, as determined by multivariate Cox regression analysis, were included in the final nomogram.

Internal validation was tested by performing 500 bootstrap resampling tests. External validation was performed by a completed independence group collected from multiple centre. Model discrimination was mainly evaluated using the C-index and area under the receiver operating characteristic curve (AUC). Calibration graphs were drawn to determine the degree of agreement between the predicted probability of a model and the observed probability. Decision curve analysis (DCA) is often used to evaluate model clinical utility.

Statistical analyses were mainly performed using X-tile software (Yale University School of Medicine, New Haven, CT, USA) and Empower (R) (www.empowerstats.com X&Y solutions, Inc. Boston, MA, USA), and R language software (version 4.2.2; Vienna, Austria; Fig. www.r-project.org).

This study was reported according to the requirements of the Transparent Reporting of a Multivariate Prediction Model for Individual Prognosis or Diagnosis (TRIPOD) statement 26.

## Results

### Baseline characteristics and survival prognosis of HCC patients

Data on 5005 pathologically confirmed cases of HCC from January 2007 to May 2012 at Sun Yat-sen University Cancer Center were collected into the training group; 882 patients met the inclusion criteria. Among them, the data of baseline figures such as PTL, albumin, TBIL, of seven patients were lost. Finally, 875 patients with intermediate-stage HCC who met the inclusion criteria were included in the training group (Figure S1). 41 patients from multiple centre were finally met the inclusion criteria and included in the validation group. All patients had hepatitis B cirrhosis-associated liver cancer. Patient baseline characteristic are shown in Table 1.

The median OS of the training patients was 22.2 months, and the 1-, 3-, and 5-year OS rates were 0.67 (95% CI 0.63-0.70), 0.38 (95% CI 0.35-0.42), and 0.29 (95% CI 0.25-0.33).

### The best cut-off value of aMAP score

**Figure 1:**
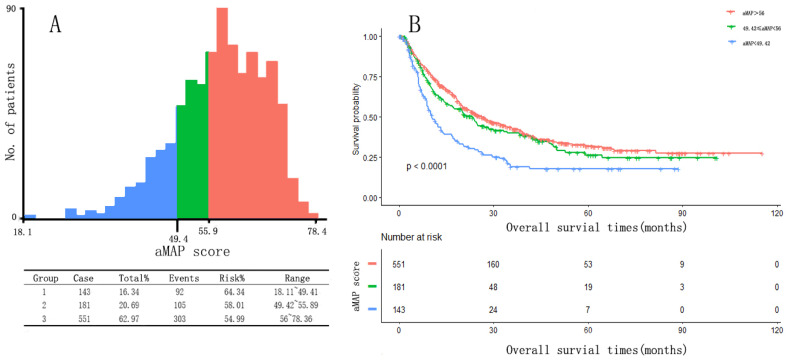
aMAP score(age-male-ALBI-platelet) cut-off values and Kaplan-Meier curve. (a) X-tile plots used to generate optimal cut-off values of aMAP score; (B) Kaplan-Meier curve for different group (aMAP ≥ 56; 49.42 ≤ aMAP < 56; aMAP < 49.42)

Two optimal cut-off points for the aMAP score were determined using X-tile: 49.42 and 56. Patients were divided into three groups with aMAP score < 49.42, 49.42 ≤ aMAP< 56, and aMAP score ≥ 56, accounting for 16.3%, 20.7%, and 63.0% of the total population. The median OS of the three groups was 10.4, 23.3 and 26.2 months. The Kaplan-Meier curve suggested that the aMAP score had a significant effect on OS (p < 0.0001) (Figure 1).

### Univariate and multivariate analyses of risk factors and OS

**Figure 2:**
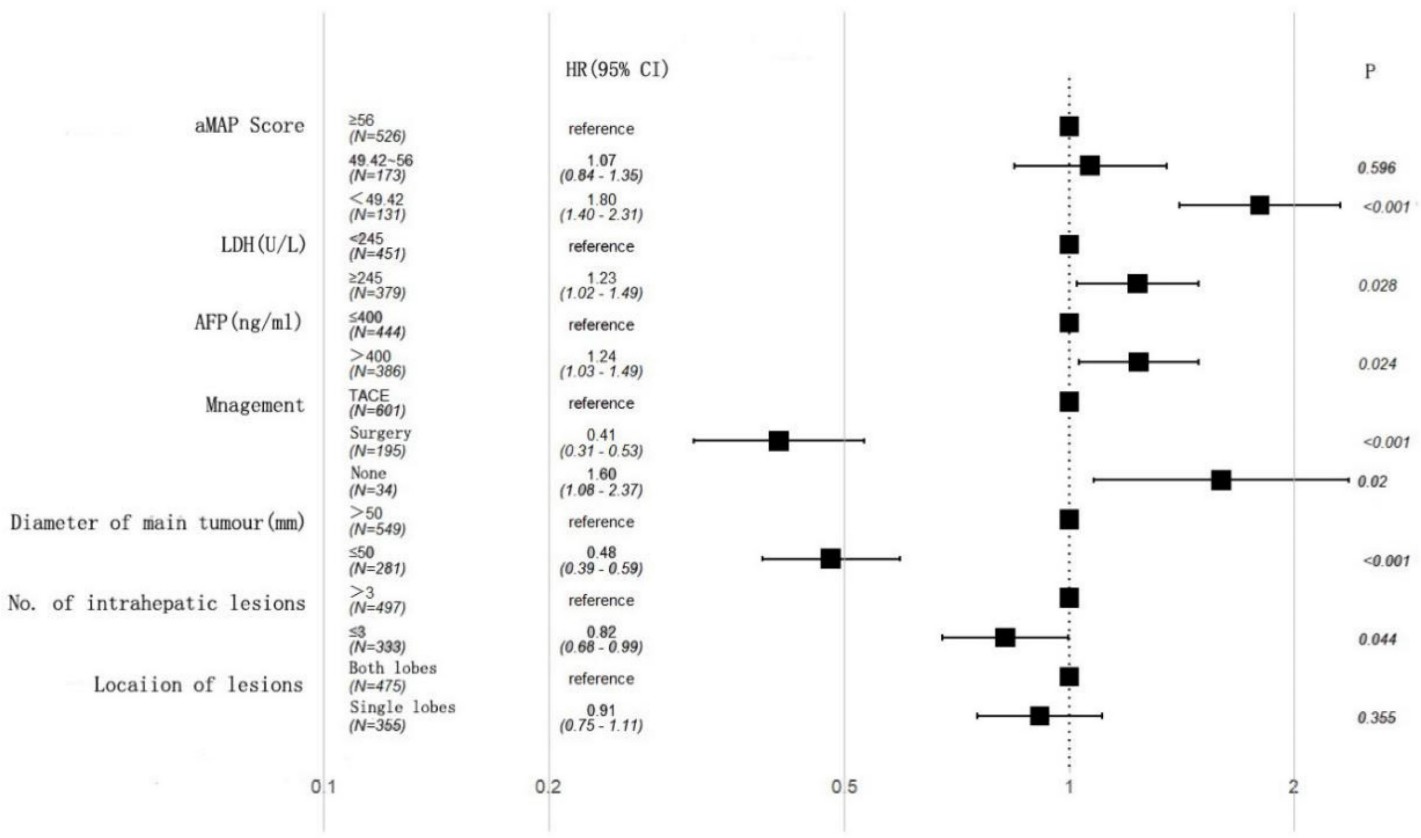
Multivariate cox regression analysis of overall survival (OS) in the training cohort.

A univariate analysis showed that AFP, lactate dehydrogenase (LDH), aMAP score, diameter of main tumor, lesion location, number of intrahepatic lesions, and treatment were associated with OS (Table 2). A multivariate analysis further demonstrated that AFP (HR: 1.24, 95% CI 1.03-1.49, p = 0.024), LDH (HR: 1.23, 95% CI 1.02-1.49, p = 0.028), diameter of main tumor (HR: 0.48, 95% CI 0.39-0.59, p < 0.001), treatment (Surgery: HR: 0.41, 95% CI 0.31-0.53, p < 0.001; None: HR: 1.60, 95% CI 1.08-2,37, p = 0.02), aMAP score (49.42-56; HR: 1.07, 95% CI 0.84-1.35, p = 0.596; <49.42; HR: 1.80, 95% CI 1.40-2.31, p < 0.001), and number of intrahepatic lesions (HR: 0.82, 95% CI 0.68-0.99, p = 0.044) were independent factors (Figure 2).

### Nomogram of OS

**Figure 3:**
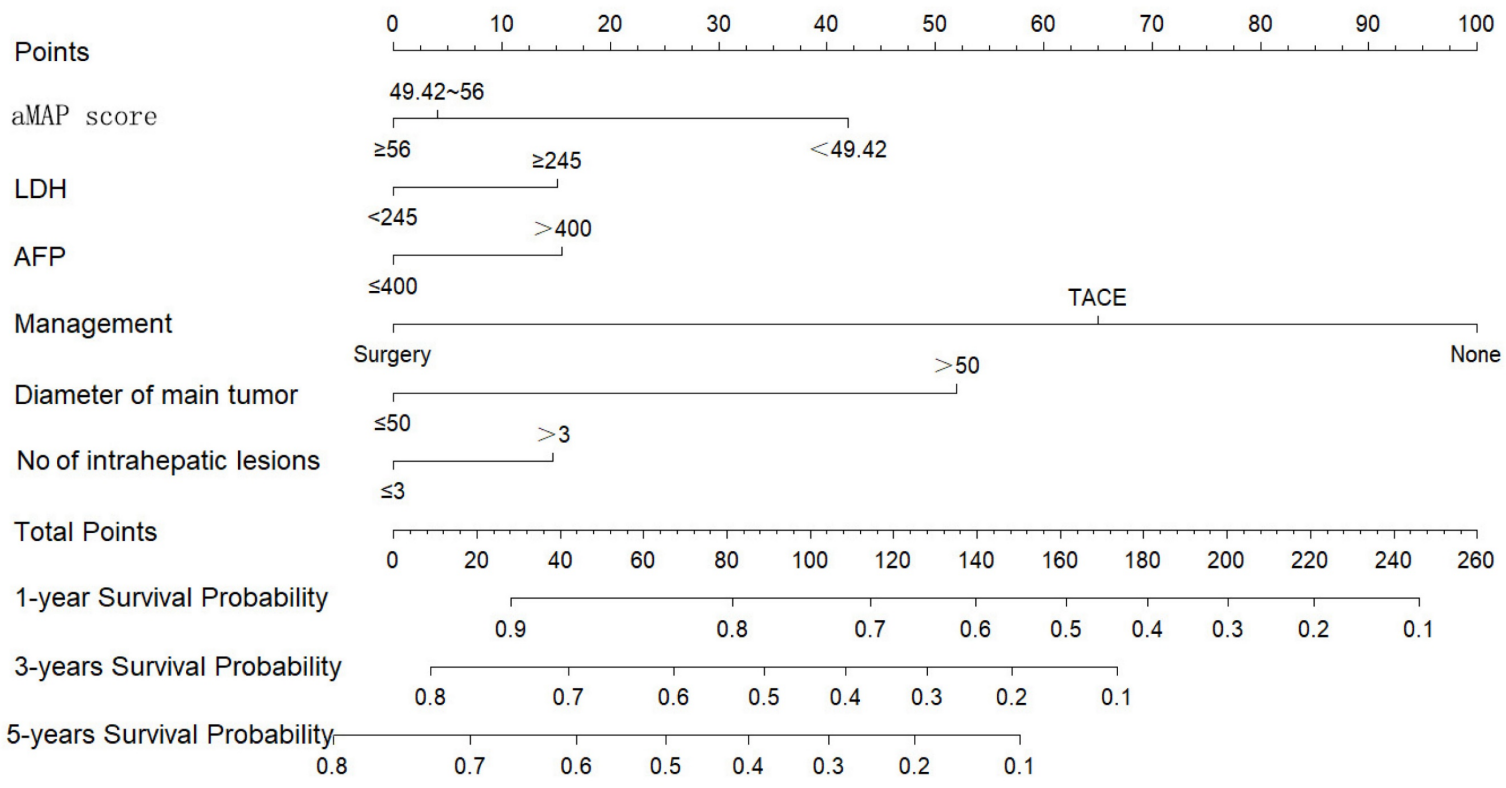
Nomogram for predicting 1, 3, and 5-year OS of intermediate-stage hepatology carcinoma (HCC) patients.

We constructed a nomogram to predict the 1-, 3-, and 5-year survival rates of intermediate-HCC patients (Figure 3). The scores for each factor were summed to provide an overall score and obtain the OS probability. For example, a patient with HCC without distant metastasis had a tumor diameter of 40 mm (0 points) at initial diagnosis, 2 intrahepatic lesions (0 points), aMAP score of 60 points (0 points), AFP of 500 ng/ml (17 points), and LDH of 246 ng/ml (16 points), and planning to receive transcatheter arterial chemoembolization (TACE) (65 points), had a total score of 98 points. The expected 1-, 3-, and 5-year OS was 75%, 45%, and 33%. We also designed calculation formulas to predict survival rates (Formula S1).

**Figure 4:**
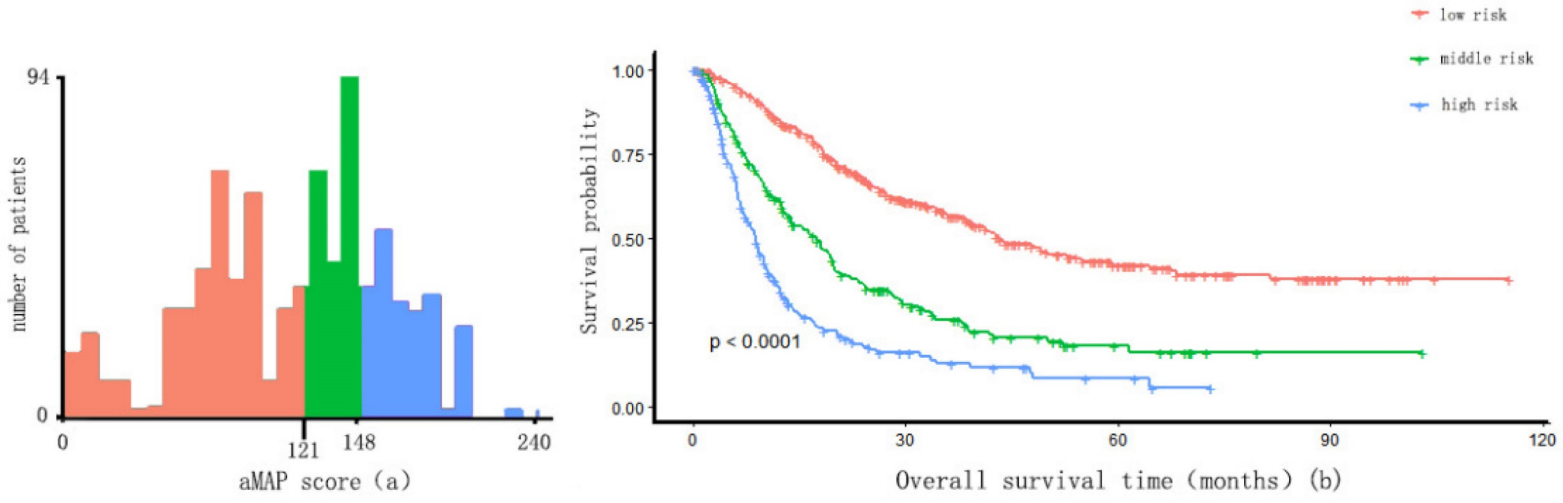
Risk score cut-off values of nomogram and Kaplan-Meier curve. (a) X-tile plots used to generate optimal cut-off values of risk score of the nomogram and divide IM-HCC into three groups (low risk: risk score ≤ 121; middle risk: 121 < risk score ≤ 148; high risk: risk score > 148); (b) Kaplan-Meier curve for different group.

Individual patient scores were calculated according to the prognostic model, and patients were divided into a low-risk group (risk score ≤ 121 points), an intermediate-risk group (121 points < risk score ≤ 148 points), and a high-risk group (risk score > 148 points) using X-tile. Significant differences were observed in the overall survival time among the three groups (p < 0.01) (Figure 4). The survival time was 43.2, 17.5, and 8.9 months in the low-risk group, intermediate-risk group, and high-risk group, respectively.

**Figure 5:**
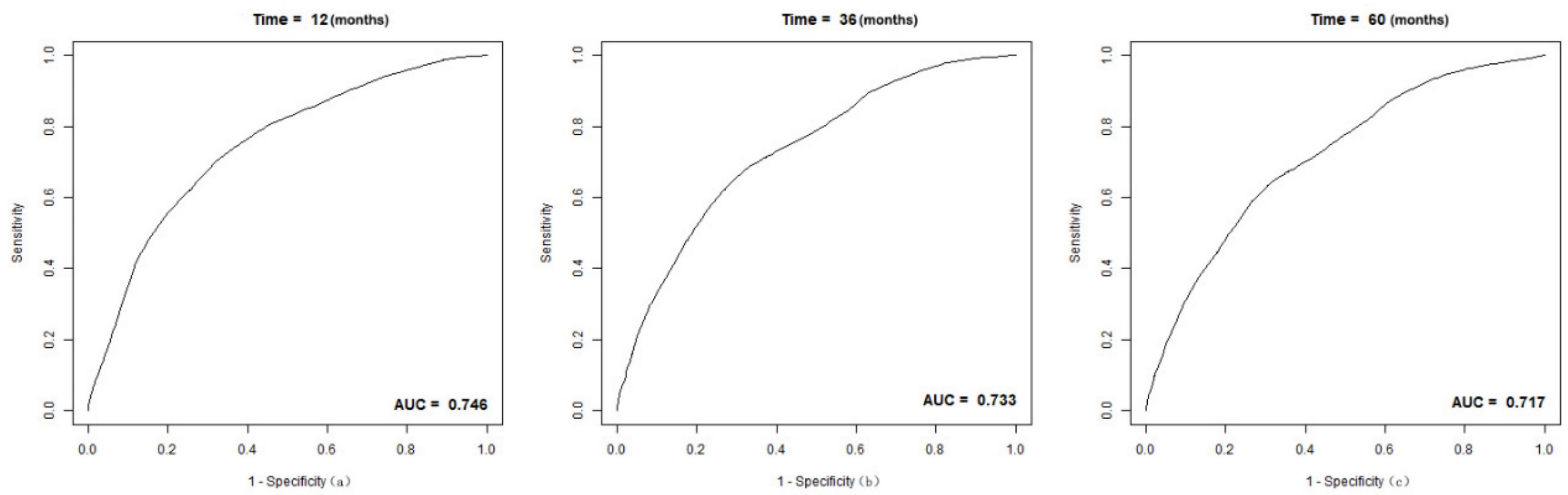
The receiver operating (ROC) curves and area under ROC characteristic curve (AUC) values for intermediate-stage HCC patient cohort: (a) 1-year OS; (b) 3-year OS; (c) 5-year OS.

**Figure 6:**
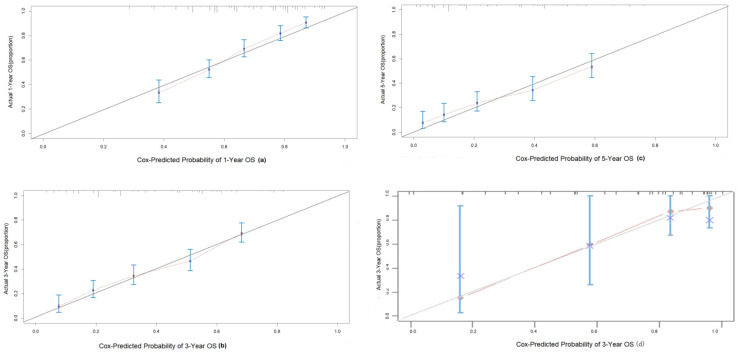
Calibration curves to predict OS in intermediate HCC patient cohort. (a) 1-year OS; (b) 3-year OS; (c) 5-year OS.

**Figure 7:**
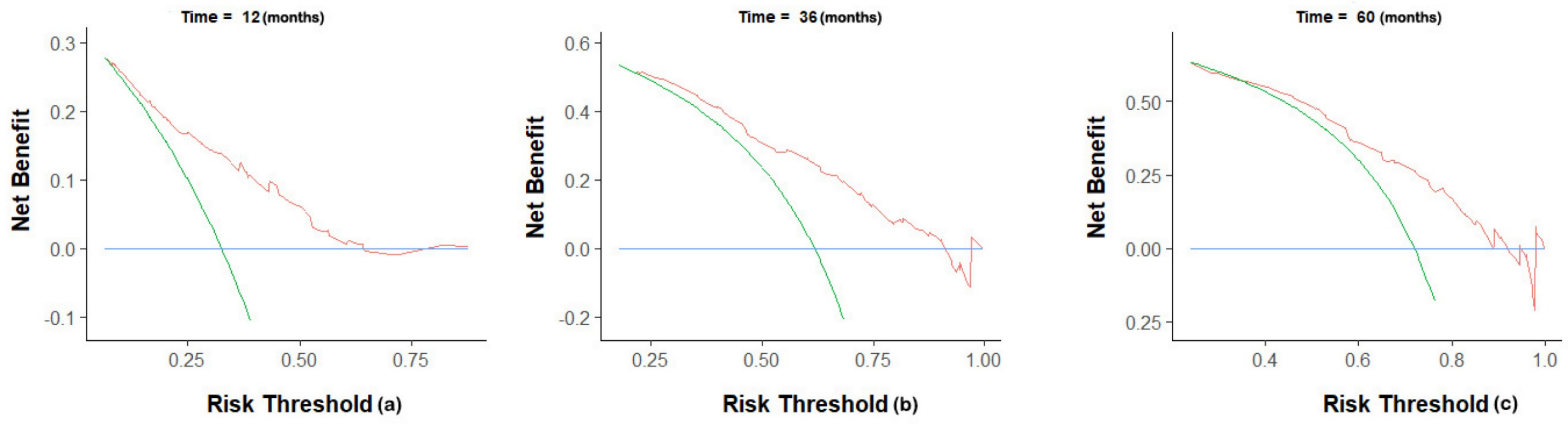
Decision curve analysis for prediction nomogram. (a) 1-year OS; (b) 3-year OS; (c) 5-year OS.

The C-index of the nomogram was 0.70(95% CI 0.68-0.72) in the training group, compared to a C-index of 0.82 in the validation group. The 1-, 3-, and 5-year AUC were 0.75, 0.73, 0.72 (Figure 5). Calibration curves showed good consistency between the actual and predicted survival rates for the prediction model (Figure 6). The clinical utility of the model was assessed using DCA (Figure 7). The intervention was supported when the predicted 1-, 3-, and 5-year overall survival rates of patients were between 0.2-0.6, 0.25-0.9, and 0.4-0.9.

## Discussion

It was widely used in HCC patients with AJCC TMN staging system world broad. However, its clinically utility is less efficient because only tumor burden was considered There is a significant difference in OS due to the heterogeneity of intermediate-stage HCC. The prognosis of HCC patients is not only affected by the size and number of tumors but also by the liver's residual function. Several models based on the liver reserve function have also been developed 27. BCLC staging is the most well-known method that provides an accurate treatment regimen and predicts a patient's prognosis. However, BCLC staging is based on the Western populations. HCC in Western populations is mostly caused by alcoholic cirrhosis, while in Asian populations, it is mostly related to viral infections, especially hepatitis B virus; thus, the performance of BCLC staging in Asian populations is not satisfactory 28. For this reason, prediction models based on Asian populations have been constructed in different countries, such as Okuda, CLIP, MESIAH, ITA.LI.CA, and HKLC score 27. These models all have a specific population and are not well applied to the whole population, especially in the interim HCC population. Based on the characteristics of interim HCC patients, we need more targeted models.

Besides the models mentioned above, researchers further established many models based on laboratory factors such as ALBI, PALBI and ALBI-T. Laboratory factors play an irreplaceable role in predicting prognosis because they show more objective and individual results. Although many different prediction models have been constructed to predict the prognosis and recurrence of HCC patients, there is still a lack of prediction models for intermediate-stage-HCC patients. Based on the characteristics of these patients, we need a more targeted model to predict survival outcomes in these patients.

Our study retrospectively analyzed data and screened six risk factors, including AFP, LDH, aMAP score, diameter of main tumor, number of intrahepatic lesions, and treatment, that affect prognosis.

AFP is a glycoprotein (approximately 70 kDa) produced by the fetal liver and yolk sac and plays an important role in the occurrence and development of liver cancer 29. Studies have found that high AFP expression in the serum or tumor cells indicates poor prognosis and is associated with vascular invasion, high tumor grade, and bulky liver cancer. High AFP concentration in serum is an independent prognostic parameter and is a more reliable prognostic predictor than AFP immunostaining of core biopsies 30. It is well known that there is a significant correlation between tumor burden and prognosis. The larger the diameter and the greater the number of tumors, the worse the prognosis. Studies have found that the incidence of microvascular invasion and intrahepatic tumor metastasis is closely correlated with tumor diameter 31. This is consistent with the conclusions of the present study.

Under anaerobic conditions, LDH can convert pyruvate into lactate for energy. A major feature of malignant tumors is hypoxia, with the tumor microenvironment being hypoxic; this affects the extracellular matrix composition, regulation of tumor immune response, and acceleration of tumor angiogenesis 32. Under anaerobic conditions, the main energy supply for tumors is lactic acid, and it has been found that tumor cells preferentially convert glucose into lactic acid for energy supply, even under aerobic conditions 33. This shift in metabolic mode favors the adaptation of tumor cells to an anaerobic environment. There is increasing evidence that poor prognosis and poor treatment response are associated with elevated LDH levels 34. Zhang et al. also found a negative correlation between the OS and LDH levels 35.

TACE is used as a routine treatment for BCLC B stage patients. However, with the advancements in medical technology, hepatectomy leads to better results in some patients. On propensity matching score analysis of 257 patients who received surgical treatment and 135 patients who received TACE for BCLC B stage, a significant difference in the OS (p < 0.001) was observed 36. The model constructed in this study also suggested that treatment was an independent risk factor for prognosis. Patients treated surgically have lower scores and better prognoses.

aMAP is a diagnostic prediction model for predicting the occurrence of liver cancer in patients with chronic hepatitis 14. The indicators included in the model mainly included age, sex, ALBI, and platelet. Age is a well-known indicator of prognosis: the prognosis of older patients is significantly worse than that of younger patients 37. ALBI is a simple, objective, evidence-based validated model for evaluating liver function and has been used internationally to predict the prognosis of patients with HCC. Platelets play a key role in the proliferation of HCC, and some studies suggest that their adhesion protein receptors GPIIb/IIIa and GPIb-IX-V may play a role in the distant metastasis of HCC 38. In addition, antiplatelet therapy was found to significantly reduce the risk of death after resection in a large retrospective study, suggesting that elevated platelets are a poor prognostic factor 39. Therefore, we conducted this study and showed that aMAP score could not only distinguish the prognosis of patients but also be used as an independent risk factor to predict the prognosis of patients.

In this study, the above factors were included in the model, and 500 internal bootstrap validations were performed to obtain a nomogram with a C-index of 0.70 (95% CI: 0.68, 0.72), in which the AUC for 1-, 3-, and 5-year OS were 0.75, 0.73, and 0.72 in the training group and a C-index of 0.82 in the validation group, which shows a good predictive ability. Meanwhile, the calibration chart of the 3-year prediction rate of the model coincides with the standard line, indicating that the OS predicted by the model is consistent with the actual. Our nomogram can accurately reflect the actual event occurrence. Moreover, our study provides a DCA curve to make a model utility evaluation index.

Our study has several strengths. First, this was a large retrospective cohort study with clinical data of 875 patients. As real-world data were used, the included population was more in line with that seen in real-world practice. Second, the nomogram we established was well performed when applied in the external group. Third, the study included the baseline characteristics of individual patients, and the differences in individual patients were fully considered. The targeted prognosis prediction for individual patients was in line with the concept of modern precision medicine. Moreover, the model was presented in the form of a nomogram, which is more convenient, intuitive, and operable in clinical settings. Patients were divided into three groups by risk score. A calculation formula based on this model was developed and designed to provide another way to predict prognosis.

However, there were some limitations. As a secondary analytical study, this study inevitably inherits the limitations of original data, including missing partial data and failure to collect more indicators that may affect the study results. At the same time, the data of this study came from a single center, which may have caused some selection bias. The selection bias may cause underestimation or overestimation of the prognosis when the model is applied to other populations.

In conclusion, aMAP score was an independent risk factor for predicting prognosis. Based on the aMAP score, we constructed a nomogram that can more objectively and accurately predict the 1-, 3-, and 5-year survival rates of individual patients with intermediate-stage HCC. Further DCA analysis indicates that our model has clinical applicability and can be useful in clinical decision-making.

## Supplementary Material

Supplementary figure and table, formula.

## Figures and Tables

**Table 1:** The Intermediate HCC Patients' Demographic

Characteristics	Total Patients (%)	
	Training group (N=875)	Validation group (N=41)
Sex		
Male	795 (90.86%)	40 (97.56%)
Female	80 (9.14%)	1 (2.44%)
Age (years)		
<55	445 (50.86%)	18 (43.90%)
≥55	430 (49.14%)	23 (56.10%)
Treatment		
TACE	632 (72.23%)	31 (75.60%)
Surgery	206 (23.54%)	6 (14.64%)
None	37 (4.23%)	4 (9.76%)
AFP (ng/ml)		
≤400	445 (50.85%)	26 (63.41%)
>400	386 (44.11%)	15 (36.59%)
NA	44 (5.02%)	0
Hgb (g/L)		
≥120	668 (76.34%)	30 (73.17%)
<120	207 (23.66%)	11 (26.83%)
PLT (*10^9/L)		
≥100	651 (74.40%)	26 (63.41%)
<100	222 (25.37%)	15 (36.59%)
NA	2 (0.22%)	0
WBC (*10^9/L)		
<11	723 (84.07%)	38 (92.68%)
≥11	137 (15.93%)	3 (7.32%)
AST (U/L)		
≥40	661 (75.54%)	25 (60.97%)
<40	214 (24.46%)	16 (39.03%)
NA	15 (1.71%)	0
LDH (U/L)		
<245	470 (53.78%)	29 (70.73%)
≥245	404 (46.22%)	12 (26.27%)
NA	1 (0.11%)	0
ALB (g/L)		
≥35	652 (74.51%)	30 (73.17%)
<35	223 (25.49%)	11 (26.83%)
TBIL (umol/L)		
≥17.1	488 (55.77%)	19 (46.34%)
<17.1	387 (44.23%)	22 (53.66%)
CRP (mg/L)		
≥10	501 (58.05%)	NA
<10	362 (41.95%)	NA
PT (s)		
<13	630 (73.34%)	27 (65.85%)
≥13	229 (26.66%)	14 (34.15%)
NA	12(1.37%)	0
Location of lesions		
Both lobes	504 (57.60%)	13 (31.70%)
Single lobe	371 (42.40%)	28 (68.30%)
Diameter of main tumor (mm)		
>50	585 (66.86%)	22 (53.66%)
≤50	290 (33.14%)	19 (46.34%)
Number of intrahepatic lesions		
>3	523 (59.77%)	30 (73.17%)
≤3	352 (40.23%)	11 (26.83%)
aMAP Score		
≥56	551 (62.97%)	17 (41.46%)
49.42≤aMAP<56	181 (20.69%)	11 (26.83%)
<49.42	143 (16.34%)	13 (31.71%)

**Table 2:** Univariate Analysis for Risk Factors of OS in Intermediate HCC patients

Variables	HR	95% CI	P
**Sex**			
Male	1		
Female	1.18	0.87-1.60	0.2780
**AFP (ng/ml)**			
≤400	1		
>400	1.43	1.20-1.71	<0.0001
**Hgb(g/L)**			
≥120	1		
<120	1	0.81-1.22	0.9765
PLT(*10^9/L)			
≥100	1		
<100	0.85	0.69-1.04	0.1149
WBC(*10^9/L)			
<11	1		
≥11	1	0.79-1.28	0.9787
**PT (s)**			
<13	1		
≥13	1.13	0.92-1.38	0.2417
**LDH(U/L)**			
<245	1		
≥245	1.56	1.31-1.86	<0.0001
**Diameter of main tumor (mm)**			
>50	1		
≤50	0.45	0.37-0.55	<0.0001
**Age (years)**			
<55	1		
≥55	0.93	0.78-1.11	0.4494
**AST (U/L)**			
≥40	1		
<40	1	0.81-1.22	0.9633
**Location of lesions**			
Both lobes	1		
Single lobe	0.69	0.57-0.82	<0.0001
**ALB (g/L)**			
≥35	1		
<35	1.30	1.07-1.59	0.0088
**CRP (mg/L)**			
≥10	1		
<10	0.86	0.72-1.02	0.0900
**aMAP Score**			
≥56	1		
49.42≤aMAP<56	1.15	0.92-1.44	0.2080
<49.42	1.85	1.47-2.34	<0.0001
**TBIL (umol/L)**			
≥17.1	1		
<17.1	1.04	0.87-1.24	0.6625
**Number of intrahepatic lesions**			
>3	1		
≤3	0.64	0.53-0.76	<0.0001
**Treatment**			
TACE	1		
Surgery	0.38	0.30-0.49	<0.0001
None	1.53	1.06-2.22	0.0245

## Abbreviations

AFP: alpha-fetoprotein
ALBI: albumin-bilirubin
aMAP: age-male-albumin-bilirubin-platelet
AUC: area under the receiver operating characteristic curve
BCLC: Barcelona Clinic Liver Cancer
CLIP: Italian Liver Cancer Program
CT: computerized tomography
DCA: decision curve analysis
HCC: hepatocellular carcinoma
LDH: lactate dehydrogenase
OS: overall survival
PALBI: platelet-albumin-bilirubin
TACE: transcatheter arterial chemoembolization
TBIL: total bilirubin
