# Supplementary material for: The aMAP Score is an Independent Risk Factor for Intermediate-stage Hepatocellular Carcinoma: A Large Retrospective Cohort Study
Source: J Cancer. 2023 May 8;14(8):1272–81. doi: 10.7150/jca.79377 (PMC10240665; doi:10.7150/jca.79377)
Supplement: Supplementary file 1 — Supplementary figure and table, formula. [file jcav14p1272s1.pdf]

Supplemental material

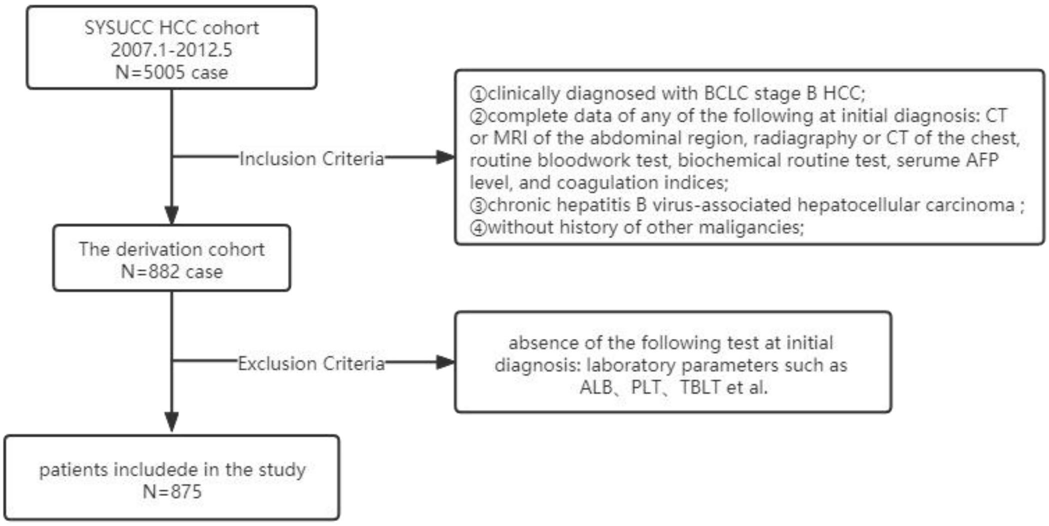

Fig. S1 Flowchart of the patient selection process in the training group.

Table S1. Score of each variable of prediction model

Variables	Group	Point
**AFP（ng/ml）**	≤400	0
	＞400	16
**LDH(U/L)**	＜245	0
	≥245	15
**Management**	Surgery	0
	TACE	65
	None	100

**Diameter of main tumour (mm)**	≤50	0
	＞50	52
**Number of intrahepatic lesions**	≤3	0
	＞3	15
**aMAP score**	≥56	0
	49.42≤aMAP＜56	4
	＜49.42	42

Formula S1. Calculation formula to predict 1-, 3-, and 5-year survival prognosis of intermediate-stage hepatocellular carcinoma patients:

$$1 - year\ survival\ probability = 5.8e^{-08} \times aMAP\ score^3 + -3.286e^{-05} \times aMAP\ score^2 + 0.001391996 \times aMAP\ score + 0.88281106$$

$$3 - year\ survival\ probability = 1.05e^{-07} \times aMAP\ score^3 + -3.4224e^{-05} \times aMAP^2 - 0.00134485 \times aMAP\ score + 0.813042628$$

$$5 - year\ survival\ probability = 1.05e^{-07} \times aMAP\ score^3 + -2.6822e^{-05} \times aMAP\ score^2 - 0.002776677 \times aMAP\ score + 0.764029545$$
